# Current status and trends in researches based on public intensive care databases: A scientometric investigation

**DOI:** 10.3389/fpubh.2022.912151

**Published:** 2022-09-15

**Authors:** Min Li, Shuzhang Du

**Affiliations:** Department of Pharmacy, The First Affiliated Hospital of Zhengzhou University, Zhengzhou, China

**Keywords:** intensive care, scientometric investigation, research, public database, status

## Abstract

**Objective:**

Public intensive care databases cover a wide range of data that are produced in intensive care units (ICUs). Public intensive care databases draw great attention from researchers since they were time-saving and money-saving in obtaining data. This study aimed to explore the current status and trends of publications based on public intensive care databases.

**Methods:**

Articles and reviews based on public intensive care databases, published from 2001 to 2021, were retrieved from the Web of Science Core Collection (WoSCC) for investigation. Scientometric software (CiteSpace and VOSviewer) were used to generate network maps and reveal hot spots of studies based on public intensive care databases.

**Results:**

A total of 456 studies were collected. Zhang Zhongheng from Zhejiang University (China) and Leo Anthony Celi from Massachusetts Institute of Technology (MIT, USA) occupied important positions in studies based on public intensive care databases. Closer cooperation was observed between institutions in the same country. Six Research Topics were concluded through keyword analysis. Result of citation burst indicated that this field was in the stage of rapid development, with more diseases and clinical problems being investigated. Machine learning is still the hot research method in this field.

**Conclusions:**

This is the first time that scientometrics has been used in the investigation of studies based on public intensive databases. Although more and more studies based on public intensive care databases were published, public intensive care databases may not be fully explored. Moreover, it could also help researchers directly perceive the current status and trends in this field. Public intensive care databases could be fully explored with more researchers' knowledge of this field.

## Introduction

Treatment of critically ill patients is one of the challenges of modern medical research. The illness severity of patients in ICUs varied from each other. Treatment decision-making of critically ill patients should be based on a large number of clinical data due to the heterogeneity of patients. However, medical workers in ICUs are too busy to collect complete data for investigations. Public databases can solve this dilemma by providing well-sorted data produced in the process of providing care for patients. Public intensive care databases are mainly multiparameter intelligent monitoring in intelligent care (MIMIC) and EICU collaborative research database (eICU-CRD) (1).

The MIMIC database is a free-access public single center database released by the Massachusetts Institute of Technology (MIT), Beth Israel Deaconess Medical Center, and Philips (2). It comprises deidentified health-related data (demography, basic signs records, medical intervention records, nursing records, imaging, and discharge records) from patients who were admitted to the ICUs of Beth Israel Deaconess Medical Center between 2001 and 2019 (3). eICU-CRD is a multi-center public intensive care database with over 200,000 admissions across the United States. Vital sign measurements, care plan documentation, diagnosis, and treatment information are collected in this database (4). Many investigations, such as evaluating the relative effectiveness of sepsis identification criteria (5) and an early prediction model of circulatory failure (6), have been conducted using public intensive care databases.

Scientometric analysis, a widely accepted research method based on statistical and visualization techniques, can depict hotspots and trends of a field. Unlike system review, scientometric investigation focuses on the metrological characteristics of literature and determines different characteristics, such as the journals, countries, institutions, authors, keywords, and references. Scientometric investigation usually includes three steps: (1) collecting data from databases; (2) analyzing data and drawing maps by software; and (3) reporting results. Several software, for example, VOSviewer and CiteSpace, have been developed for scientometric investigations. Lu et al. summarized the key topics through scientometric investigation of publications related to peptide receptor radionuclide therapy using VOSviewer and CiteSpace (7). The two user-friendly software allow beginners to know about the current status of a specific field and identify hotspots with ease.

Many studies based on public intensive care databases have been published. However, there is no in-depth analysis of the cooperation network, research hotspots, and trends of this field. This study presented a comprehensive view of the studies based on public intensive care databases, such as the number of publications per year, key journals, productive countries, institutions, and authors. We hope our study can promote the full utilization of public databases that are beneficial to researchers, clinicians, and patients.

## Materials and methods

### Data source and search strategy

Software could not analyze data from different databases at the same time. We retrieved English literatures from the Web of Science core Collection (WoSCC) in our study instead of searching other databases, such as Pubmed or Dimensions. Search strategies were [“eICU-CRD” (All Fields) OR “eICU Collaborative Research Database” (All Fields)] or [“Multiparameter Intelligent Monitoring in Intensive Care” (All Fields) OR “Medical Information Mart for Intensive Care” (All Fields)]. We selected “article” or “review” as the document types and excluded letters, news, conference summaries, and other documents. Retrieval time was limited from 2001 to 2021.

### Statistical analysis

The quality of the journal (IF2021 and JCR) were obtained from 2021 Journal Citation Reports (JCR) (Clarivate Analytics, Philadelphia, USA). VOSviewer (1.6.18) was used to identify productive journals and co-cited journals, countries, institutions, and authors and visualize cooperation networks. In the VOSviewer network maps, nodes represent elements, such as countries, institutions, and authors. The size of the nodes represents the number of publications or occurrence frequency. The shorter distance between two nodes, the closer cooperation between two elements. The color of the nodes represents publishing time. The thickness of links represents cooperation intensity between two elements. The color of the links represents the first year of cooperation. Cold colors represent earlier years, while warm colors represent recent years. We used CiteSpace (5.8.R3) to conduct keyword clustering and detect the references with strong citation burstness to identify hot topics. Data were managed using Microsoft Office Excel 2019 (8).

## Results

### Annual growth trend of publications

**Figure 1:**
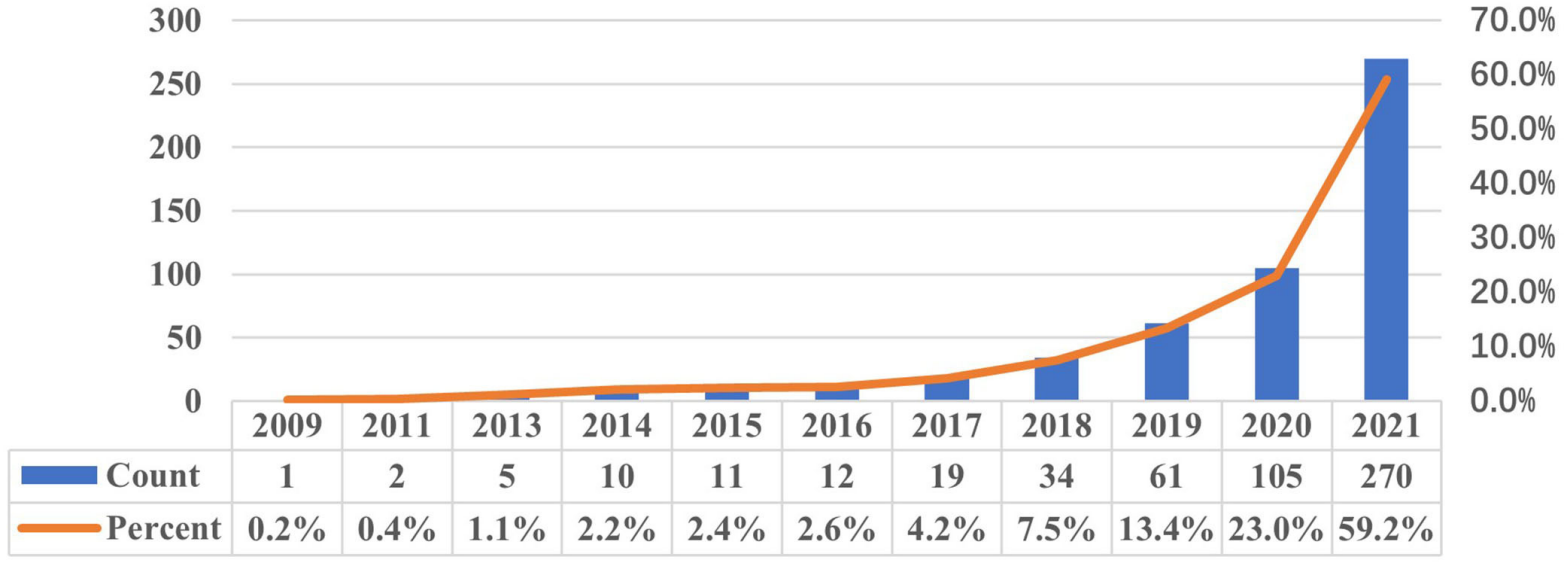
The number of publications per year (2009–2021).

In total, 606 publications were finally included for analysis. The number of publications published based on public intensive care databases from 2009 to 2021 was shown in Figure 1. Publications based on public intensive care databases were published every year since 2009. The number of annual publications represented a significant upward trend during the investigation period. The annual publication number was 12 (2.6%) in 2016, 34 (7.5%) in 2018, and 270 (59.2%) in 2021.

### Journals and co-cited academic journals

A total of 244 journals have published literatures based on public intensive care databases. The top 10 journals account for 30.7% (186/606) of the total publications included in this study (Table 1). There were eight journals with more than 10 publications, of which *Frontiers in Medicine* (IF2021 = 5.058, Q2) was the most productive journal with 30 publications, followed by the *International Journal of General Medicine* (IF2021 = 2.145, Q3), *Critical Care* (IF2021 = 19.346, Q1), and scientific reports (IF2020 = 4.996, Q2).

**Table 1:** Top 10 journals in terms of publications and top 10 journals in terms of co-citations.

**Rank**	**Journal**	**Count**	**IF2020**	**JCR**	**Co-cited journal**	**Count**	**IF2021**	**JCR**
1	Front Med	30	5.058	Q2	Crit Care Med	1161	9.296	Q1
2	Int J Gen Med	29	2.145	Q3	Crit Care	641	19.346	Q1
3	Crit Care	18	19.346	Q1	Jama-J Am Med Assoc	592	157.335	Q1
4	Sci Rep	18	4.996	Q2	Intens Care Med	582	41.787	Q1
5	Bmj Open	17	3.017	Q2	Sci Data	485	8.501	Q1
6	JMIR Med Inf	17	3.231	Q3	New Engl J Med	349	176.079	Q1
7	J Am Med Inform Assn	16	7.942	Q1	PLoS ONE	276	3.752	Q2
8	Front Cardivovasc Med	15	5.846	Q2	Chest	269	10.262	Q1
9	Ann Transl Med	14	3.616	Q3	Circulation	242	39.918	Q1
10	PLoS ONE	12	3.752	Q2	J Am Med Inform Assn	240	7.942	Q1

Two journals have a co-citation relationship when they are cited simultaneously in one or more publications. Among 14,054 co-cited academic journals, 10 journals had co-citations over 4,837 times. *Crit care med* had the most co-citations (IF2020 = 9.296, Q1), followed by *Intensive care medicine* (IF2021 = 19.346, Q1), and *JAMA-journal of the American medical association* (IF2020 = 157.335, Q1) (Table 1).

### Country and institution analysis

A total of 48 countries have published literature based on public intensive care databases, mainly China (336), the United States (199), England (37), Canada (26), and Australia (19) (Table 2).

**Table 2:** Top 10 countries and institutions in terms of the publication number.

**Rank**	**Country**	**Count**	**Institution**	**Count**
1	China	336	MIT (USA)	43
2	USA	199	Zhejiang Univ (China)	40
3	England	37	Sun Yat Sen Univ (China)	36
4	Canada	26	Beth Israel Deaconess Med Ctr (USA)	32
5	Australia	19	Wenzhou Med Univ (China)	28
6	India	13	Xi'an Jiao Tong Univ (China)	28
7	South korea	13	Ji Nan Univ (China)	18
8	Germany	12	Cent South Univ (China)	17
9	Italy	12	Chinese Peoples Liberat Army Gen Hosp (China)	14
10	Singapore	11	Beth Israel Deaconess Med Ctr (USA)	13

**Figure 2:**
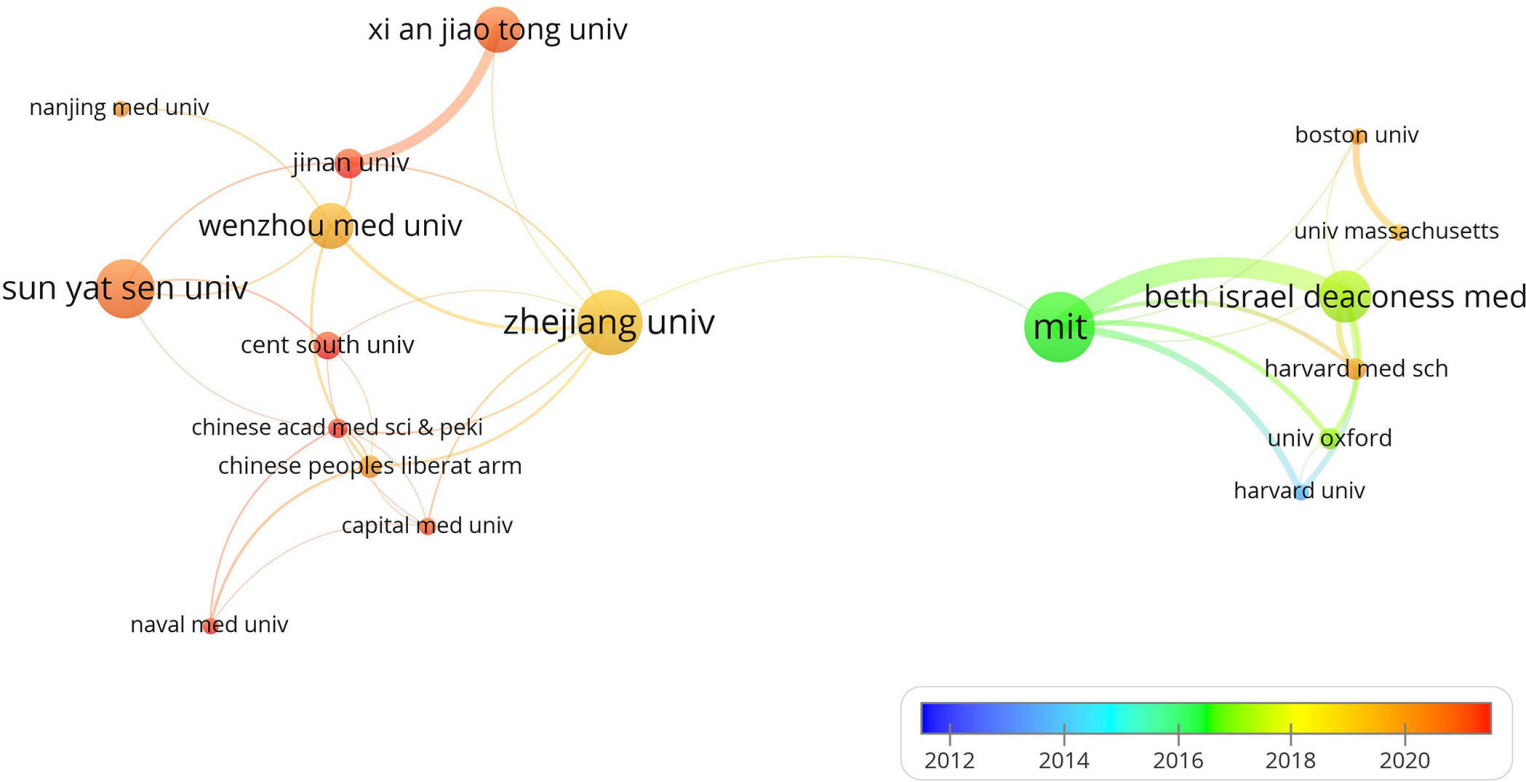
The network of co-institute.

A total of 713 institutions have published literature based on public intensive care databases. MIT had the largest number of publications with 43, followed by Zhejiang University (40) and Sun Yat-sen University (36) (Table 2). Institutions with the more than eight publications were included to construct the co-institution map (Figure 2). MIT and Zhejiang University occupied important positions in the network of institute cooperation. According to the color of the links, institutions began to cooperate frequently since 2016. It may due to the release of MIMIC-III in 2016 which drew the attention of researchers around the world (3).

### Author analysis

**Figure 3:**
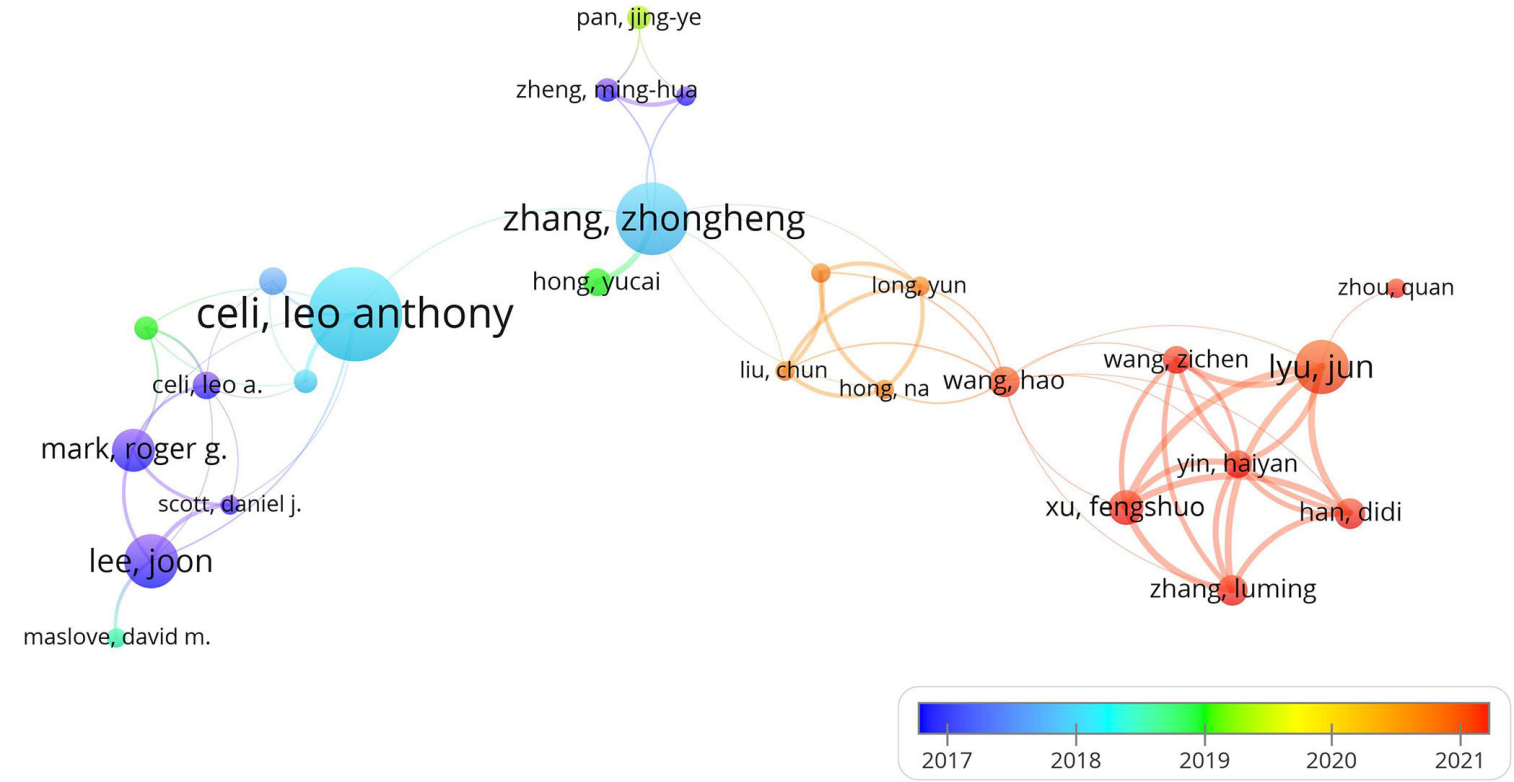
The network of co-authorship.

A total of 2,638 authors have published literature based on public intensive care databases. Top 10 authors have published 129 (21%) articles (Table 3). Leo Anthony Celi of MIT published the most articles (*n* = 25), followed by Zhongheng Zhang (*n* = 19) and Lee Joon (*n* = 14). Authors with the more than 6 publications were included to construct the co-author map. Leo Anthony Celi, who had participated in the release of MIMIC-III database, cooperated closely with researchers in this field. Zhang Zhongheng has closer cooperation with Chinese authors (according to the name of authors). However, the top two authors only jointly published one article based on the MIMIC database (9). Most of Chinese authors published literatures in recent years (Figure 3).

**Table 3:** Top 10 authors in terms of the publication number.

**Rank**	**Author**	**Publications**
1	Celi, Leo Anthony	25
2	Zhang, Zhongheng	19
3	Lee, Joon	14
4	Lyu, Jun	14
5	Mark, Roger G.	11
6	Mcmanus, David D.	9
7	Xu, Fengshuo	9
8	Bashar, Syed Khairul	8
9	Han, Didi	8
10	Luo, Yuan	8

### Keywords analysis

Co-occurring keywords reflect hotspots of studies based on public intensive care databases. Keywords with high frequency are listed in Table 4. Excluding keywords (intensive care unit, critically ill patient, management, and system) lacking guiding significance, “mortality,” “machine learning,” “sepsis,” “acute kidney injury,” “prognosis,” “deep learning,” “nomogram,” “prediction,” “hospital mortality,” and “mortality prediction” are the top 10 keywords with high frequency. A total of 15 keyword clusters were obtained by CiteSpace-6.1.2. Keyword clustering is the clustering of closely linked keywords from which you can see Research Topics form in a certain field (10). The top 9 clusters and keywords included in each cluster are listed in Table 5. “Acute respiratory distress syndrome,” “Albumin ratio,” “Arterial blood pressure,” “External validation,” “To-albumin ratio,” “Retrospective study,” “Bayesian filter,” “Patient profiles,” “Treatment-related complications,” and “Prediction” are potential main topics in this field.

**Table 4:** Top 20 keywords with high frequency.

**Keywords**	**Strength**	**Freq**	**Keywords**	**Strength**	**Freq**
Mortality	104	112	Natural language processing	14	11
Machine learning	108	79	Atrial fibrillation	13	10
Sepsis	102	76	Lactate	18	10
Acute kidney injury	62	45	All-cause mortality	8	9
Prognosis	33	30	Data mining	8	9
Deep learning	35	27	Medical informatics	16	9
Nomogram	43	24	Mechanical ventilation	9	8
Prediction	32	19	Prediction model	19	8
Hospital mortality	20	16	Propensity score matching	16	8
Mortality prediction	15	14	Septic shock	8	8

**Table 5:** Top 9 clustering and keywords included in each cluster.

**ID**	**Cluster**	**Size**	**Keyword included**
0	Acute respiratory distress syndrome	45	Prediction; hospital; mortality; APACHE; BMI
1	Albumin ratio	44	Septic shock; lactate; criteria; serum albumin
2	Arterial blood pressure	38	Model; algorithm; pharmacovigilance; morbidity
3	External validation	32	Sepsis; acute kidney injury; validity; patient readmission
4	To-albumin ratio	29	Score; heart failure; complication; data element
5	Retrospective study	27	Impact; risk factor; mechanical ventilation; neutrophil
6	Bayesian filter	24	Blood pressure; feature extraction; electrocardiogram; blood pressure estimation
7	Patient profiles	22	Admission; electronic health record; length of stay; arterial pressure
8	Treatment-relatedcomplications	16	Cost; anemia; prednisone; clostridium difficile infection
9	Prediction	3	Spinal anesthesia; heart rate variability; elective cesarean delivery

### References with citation burst

“Citation burst” refers to a body of literature that is frequently cited over a period of time. References with a high citation burstness can, to a certain extent, reflect the emerging trends or topics within a field (10). The minimum duration of burst was set at 2 years. γ was set as 0.5. Table 6 lists top 17 references with the strongest citation bursts. The red line in the blue line represents the time interval of citation burst. The longest burst among the 17 references was “Multiparameter Intelligent Monitoring in Intensive Care II: A public-access intensive care unit database” (2). Nine references ended before 2018. Jia et al. explored risk factors for acute respiratory distress syndrome in patients mechanically ventilated, which brought a burst of study on risk factors (11). Abhyankar et al. developed a generalizable method for identifying patient cohorts from electronic health records by combining structured and unstructured data, which were of great benefit for identifying a large set of patients for investigations (12). Article entitled “Toward Ubiquitous Blood Pressure Monitoring *via* Pulse Transit Time: Theory and Practice” (13) and article entitled “Clinical Practice Guideline for the Evaluation and Management of Chronic Kidney Disease” (14) indicated that more methods and types of diseases are investigated. References related to machine learning and in-depth learning (5, 15) burst after 2019 and lasted until retrieval time, which manifested that machine learning was the frontier of studies based on public intensive care databases.

**Table 6:** Top 17 references with the strongest citation bursts.

**References**	**Begin**	**End**	**2004–2021**
Saeed (16), Comput Cardiol	2008	2010	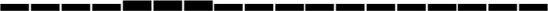
Saeed Mohammed (2006), AMIA Annu Symp Proc	2010	2011	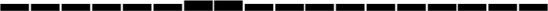
Li (2008), Physiol Meas	2008	2013	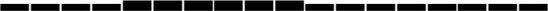
Aboukhalil (2008), J Biomed Inform	2010	2016	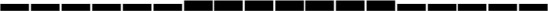
Jia (2008), Chest	2011	2016	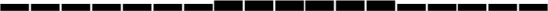
Scott (2013), BMC Med Inform Decis,	2014	2016	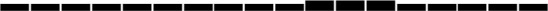
Lehman Li-wei (17), AMIA Annu Symp Proc	2015	2016	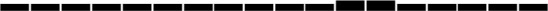
Lee (2011), IEEE Eng Med Bio	2015	2016	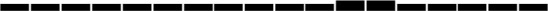
Zhang (2014), J Thorac Dis	2015	2016	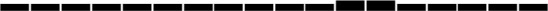
Abhyankar (2014), J Am Med Inform Assn	2016	2017	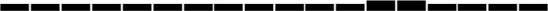
Abhyankar (2012), Crit Care	2016	2017	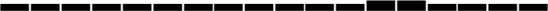
Saeed (2), Crit Care Med	2012	2018	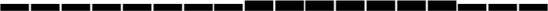
Seymour (18), JAMA-J Am Med Assoc	2017	2018	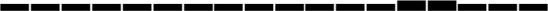
Mukkamala (2015), IEEE T Bio-Med Eng	2018	2019	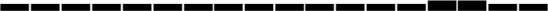
Eckardt (2012), Kidney Int Suppl	2019	2021	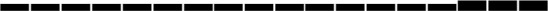
Rajkomar (2018), NPJ Digit Med	2019	2021	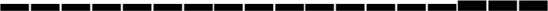
Desautels (2016), JMIR Med Inf	2019	2021	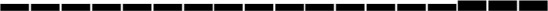

## Discussion

### Basic information

A total of 606 articles in 244 journals with 14,054 co-cited references by 713 institutions from 48 countries were included in our study. Articles published in this field could be divided into two stages. Before the release of the MMIC-III database in 2016, only a few articles were published each year. After 2016, the number of articles published increased year by year. An upward trend suggested that studies based on public intensive care databases have received increasing attention in recent years. Moreover, according to the color of the links and nodes of author analysis, many Chinese articles were published in the last 2 years, indicating that public intensive care databases had attracted the attention of Chinese researchers. Critical Care Medicine and JAMA-Journal of the American Medical Association, which are journals with high academic influence, ranked the first and second in the co-cited journals. Meanwhile, the IF2021 of most productive journals is lower than 5, indicating the relatively low quality of publications in this field. Analysis results of institution, country, and author are consistent. For example, Zhang Zhongheng from Zhejiang University (China) and Leo Anthony Celi from MIT (USA) occupied important positions in the research based on public intensive care databases. It is worth noting that the cooperation between institutions in the same country is closer. Strengthening international cooperation may improve the quality of studies based on public intensive care databases.

### Research topics

Keyword clustering is the clustering of closely linked keywords from which you can see Research Topics forming in a certain field. According to the cluster name and included keyword, six areas are summarized by reading the full text of included literature in the cluster. According to the result of keyword clustering, six hot topics could be roughly summarized after referring to included literature: (1) prediction of mortality, serious complications, and readmission of critically ill patients; (2) exploration of risk factors of mortality and prognosis of critically ill patients; (3) studies on vital signs of critically ill patients; (4) scoring system and external verification of standards or guidelines; (5) impact of treatments and drugs on outcomes of critically ill patients; and (6) introduction of databases and data processing methods.

Machine learning was used in the prediction of mortality, adverse prognosis, and morbidity of critically ill patients. The condition of patients in intensive care units changes rapidly. The prediction of serious complications or nosocomial diseases, such as acute kidney injury, pressure injury, and anemia, was conducive to taking preventive measures as soon as possible. Goodwin et al. developed a generalizable model capable of leveraging clinical notes to predict three nosocomial diseases 24–96 h in advance (19). Fialho et al. selected commonly texted variables, such as heart rate, temperature, platelets, non-invasive arterial blood pressure, spO_2_, and lactic acid during the last 24 h before discharge (20). The collection of these variables selected is not complex in ICUs. Meanwhile, they were capable of predicting ICU readmissions and providing support for clinicians to make plans to reduce the risk of readmission.

One main subject of research based on public intensive care databases was to analyze whether some factors affected the prognosis or mortality of critically ill patients. Studies mainly focused on acute respiratory distress syndrome, sepsis shock, acute renal failure, intracerebral hemorrhage, and myocardial infarction (21–25). Factors analyzed in studies were lactic acid, body mass index (BMI), red blood cell distribution width (RDW), neutrophil to lymphocyte ratio, blood oxygen saturation, anion gap, and coagulation variables (26–31).

Studies on the vital signs of patients focused on estimating blood pressure, heart rate, and respiratory rate, reducing the probability of false alarms and detecting noise in electrocardiogram. For example, Chon et al. developed an automated method to detect noise from long-term electrocardiogram signals recordings in the MIMIC III database. This detection algorithm could accurately detect the presence of atrial fibrillation with only 5.7% false positives (32).

External verification of scoring systems, criteria, or guidelines is one of the applications of public intensive care databases. A number of cases were included in public databases that could be used to verify diagnostic criteria. Controversy existed in the diagnostic criteria of sepsis. Retrospective study conducted by Xueling Fang verified that sepsis-3 diagnostic criteria narrow the sepsis population at the expense of sensitivity, which may delay disease diagnosis (33). The modified Nutrition Risk in the Critically ill (mNUTRIC) score was introduced to evaluate the nutritional risk of patients in the ICU (34). Zheng et al. investigated the prediction value of mNUTRIC score of patient in cardiothoracic surgery recovery unit in the MIMIC database (35).

Exploring the effects of treatment is an emerging topic of studies based on public databases. Su et al. used the MIMIC database to select strategy for sedation depth in critically ill patients using a machine learning model (36). The efficacy and safety of loop diuretic use in critically ill patients on vasopressor support or in shock remain unclear. The relationship between loop diuretic use and hospital mortality in critically ill patients with vasopressor support was studied using data extracted from the MIMIC database. Results showed that loop diuretic use was associated with lower mortality without an obvious compromise in the mean arterial pressure (37).

Some of the included articles were about the introduction of databases, data processing, and data extraction methods. Public intensive care databases are highly susceptible to quality issues, such as missing information and erroneous data due to a large number of parameters. Venugopalan et al. used a MIMIC database as an example to demonstrate new imputation techniques for each type of missing data. The novel imputation techniques outperformed standard mean filling techniques in predicting ICU mortality (38). Rich information in clinical narratives can help to detect adverse drug events (ADEs) since details of the diseases (such as, signs and symptoms, disease status, and severity) are all typically recorded in clinical text. Deep learning methods were utilized to recognize drug names, attributes entities, and relations from clinical narratives in the MIMIC database. In comparison with traditional machine learning algorithms, it could simultaneously recognize entities of ADEs, the reason, and their relations with medications (39).

### Strengths and limitations

Our study has several strengths. First, this is the first study to provide a comprehensive insight into the status, hotspots, and trends of research on public intensive care databases using the scientometric analysis. Second, CiteSpace and VOSviewer are widely used tools for scientometric analysis, which assures the reliability of results. Third, compared with system reviews, scientometric analysis is relatively more objective and comprehensive.

This is the first time that scientometrics has been used in the investigation of studies based on public intensive databases. This is different from other conventional scientometric investigations which reported the status of a certain field (a disease or a drug). Although more and more studies based on public intensive care databases were published, public intensive care databases may not be fully explored. Research using public data is mainly explored with a few countries, institutions, and researchers. As shown in the country analysis, studies based on public intensive care databases mainly in the United States and China (88% of included literature). According to the analysis of institutions and authors, Zhongheng Zhang from Zhejiang University and Celi Leo Anthony (14% of included literature) from MIT were key authors. Moreover, research topics were narrow, with only mainly six directions. Two of the six topics are related to the prediction of the outcome. Disease types explored are mainly limited to sepsis, acute respiratory distress syndrome, heart failure, and acute kidney injury. Judging from the published journals, quality of the studies based on intensive care databases is not high since most of the journals ranked as Q3 in JCR.

Our results can also help new researchers interested in this field quickly to get status, hotspots, and trends in this field. They can know which researchers or institutions to learn from and carry out studies quickly (top 2 authors). Database developers can know whether the databases are fully utilized as they imagine. If not, they can find out the reasons and make some improvements. For example, there is no index table for drug names, which makes studies evaluating the efficacy of drugs difficult to carry out. In addition, there are no images in the databases, which makes the machine learning studies based on original image impossible to carry out. Although public intensive care databases have been explored by more and more researchers (the number of publications in 2021 accounted for half of the total number of publications), the quality of journals is not high. This may be because studies based on intensive care databases can only do retrospective studies. Studies based on large amount of data in databases are valuable and should be recognized by editors. Studies based on data collected from on real clinical diagnosis and treatment without intervene are valuable for clinicians. Researchers should improve the quality of their literature by taking measures, such as working on more valuable questions and using scientific statistical methods.

Our study still has some limitations. First, our chosen software and data expertise did not allow us to combine data from different sources for the analysis shown here. This would have been valuable as it could have extended our analysis to a wider dataset. However, as argued in our search strategy section, for the statements that we make in our article on large scale trends, we feel that our sample size is large enough to make robust comments of the type given here. The main shortcoming of our approach is that geographic diversity may not be fully represented (due to the English-centric nature of WoS) (7, 8).

## Conclusion

To our knowledge, this is the first scientometric investigation for studies based on public intensive care databases. Our study provided an overview of research hotspots, trends, key journals, authors, institutions, countries, and their co-operative relations. China contributed the most in the studies based on public intensive care databases. MIT and Zhejiang University, occupied important position in the network of institute cooperation. Leo Anthony Celi of MIT and Zhongheng Zhang of Zhejiang University had the highest number of articles and also cooperated most closely with other authors. The number of studies based on public intensive care databases increased quickly after 2018 with more and more disease, medicines, and research methods being explored by more researchers. Six main topics were summarized through keywords analysis. Until now, the research method of machine-learning is commonly used research method. This scientometric investigation could help researchers directly perceive the current status and trends in this field. As more and more researchers know about public databases, public intensive care databases will be fully-explored and promote the development of critical care medicine.

## Data availability statement

The original contributions presented in the study are included in the article/supplementary material, further inquiries can be directed to the corresponding author/s.

## Author contributions

ML and SD equally contributed to the conception and design of the research. ML contributed to the collection of literature, the acquisition, analysis, and interpretation of the data. Both authors drafted the manuscript, critically revised the manuscript, agree to be fully accountable for ensuring the integrity and accuracy of the work, read, and approved the final manuscript.

## Funding

This work was supported by the grant received from Funding: Health Commission of Henan Province LHGJ20190278.

## Conflict of interest

The authors declare that the research was conducted in the absence of any commercial or financial relationships that could be construed as a potential conflict of interest.

## Publisher's note

All claims expressed in this article are solely those of the authors and do not necessarily represent those of their affiliated organizations, or those of the publisher, the editors and the reviewers. Any product that may be evaluated in this article, or claim that may be made by its manufacturer, is not guaranteed or endorsed by the publisher.
